# Venous cerebral blood flow quantification and cognition in patients with sickle cell anemia

**DOI:** 10.1177/0271678X211072391

**Published:** 2022-01-06

**Authors:** Hanne Stotesbury, Patrick W Hales, Melanie Koelbel, Anna M Hood, Jamie M Kawadler, Dawn E Saunders, Sati Sahota, David C Rees, Olu Wilkey, Mark Layton, Maria Pelidis, Baba PD Inusa, Jo Howard, Subarna Chakravorty, Chris A Clark, Fenella J Kirkham

**Affiliations:** 1 Developmental Neurosciences, UCL Great Ormond St. Institute of Child Health, London, UK; 2 Division of Psychology and Mental Health, Manchester Centre for Health Psychology, University of Manchester, Manchester, UK; 3 Radiology, Great Ormond Hospital for Children NHS Trust, London, UK; 4 King’s College London, London, UK; 5 North Middlesex University Hospital NHS Foundation Trust, London, UK; 6 Haematology, Imperial College Healthcare NHS Foundation Trust, London, UK; 7 Department of Haematology and Evelina Children’s Hospital, Guy’s and St Thomas’ NHS Foundation Trust, London, UK

**Keywords:** Cerebrovascular disease, hematology, hemodynamics, cognition, MRI

## Abstract

Prior studies have described high venous signal qualitatively using arterial spin labelling (ASL) in patients with sickle cell anemia (SCA), consistent with arteriovenous shunting. We aimed to quantify the effect and explored cross-sectional associations with arterial oxygen content (CaO_2_), disease-modifying treatments, silent cerebral infarction (SCI), and cognitive performance. 94 patients with SCA and 42 controls underwent cognitive assessment and MRI with single- and multi- inflow time (TI) ASL sequences. Cerebral blood flow (CBF) and bolus arrival time (BAT) were examined across gray and white matter and high-signal regions of the sagittal sinus. Across gray and white matter, increases in CBF and reductions in BAT were observed in association with reduced CaO_2_ in patients, irrespective of sequence. Across high-signal sagittal sinus regions, CBF was also increased in association with reduced CaO_2_ using both sequences. However, BAT was increased rather than reduced in patients across these regions, with no association with CaO_2_. Using the multiTI sequence in patients, increases in CBF across white matter and high-signal sagittal sinus regions were associated with poorer cognitive performance. These novel findings highlight the utility of multiTI ASL in illuminating, and identifying objectively quantifiable and functionally significant markers of, regional hemodynamic stress in patients with SCA.

## Introduction

Sickle cell anemia (SCA) is the most common single gene disorder worldwide. The disorder is associated with neurological complications, including overt stroke, silent cerebral infarction (SCI), and cognitive impairment,^1^ all of which may significantly impact social and economic mobility, and quality of life.^2^ Recent evidence points to a role for cerebral hemodynamic stress in neurological complications in SCA but cerebral hemodynamics are complex and remain relatively poorly understood.

As has been described in other vascular beds,^3^ the cerebral circulation may exhibit a “perfusion paradox” in SCA, with hyperperfusion in the macrocirculation, and relative hypoperfusion in the microcirculation.^1^ As in other anemias, cardiac output is increased,^4^ with downstream increases in global gray matter CBF widely and consistently observed.^5^ Increases in global gray matter CBF are associated with reduced arterial oxygen content,^6^ indicative of a compensatory mechanism, which appears to maintain oxygen delivery when averaged globally.^7^ Despite this, ischemic tissue injury, including SCI and reduced integrity, reportedly frequently occur in the deep watershed white matter, a region that is supplied by long perforating and often tortuous terminal branches.^1,8,9^ Deep white matter CBF is therefore inherently low, and oxygen delivery may be disproportionately reduced in these regions in patients with SCA,^7^ perhaps secondary to red blood cell sickling, arteriovenous shunting, and/or an exhaustion of compensatory mechanisms. Reduced cerebrovascular reserve (CVR)^10–14^ and abnormal oxygen extraction fraction (OEF) have also been reported in patients with SCA, along with potential downstream effects on white matter tissue integrity.^15,16^ There are reports of both higher^17–19^ and lower global OEF^20,21^ in patients with SCA, depending in part on data calibration model, with consensus on model validity still developing.^22^

Increased global gray matter CBF has been confirmed using various methods in patients with SCA, including single inflow-time (TI) arterial spin labelling (ASL). SingleTI ASL involves magnetically labelling protons in arterial blood before entering a region of interest (ROI) by the application of inversion pulses at a slice below the ROI. After a few seconds (TI), the magnetically labelled protons flow into the ROI, where they interact with those in tissue, reducing net magnetisation. An image is then acquired, and subtracted from a control image with no labelled protons. SingleTI ASL can be used to measure blood flow in the microvasculature, by acquiring images at a sufficiently long TI, such that the majority of the labelled protons have passed through the larger feeding arteries and reached the microvasculature. However, estimating the distribution of labelled water between the vascular and tissue compartments is challenging with ASL, although two-compartment modelling has made some progress in this area.^23,24^

Recent singleTI ASL studies in patients with SCA have observed abnormally high signal in the sagittal sinus,^25,26^ a potential marker of arteriovenous shunting. In healthy populations, ASL labeled blood water is believed to exchange with tissue water at the microcirculatory level. The T1 decay of the labeled blood water (T1_bl_) is believed to be shorter than or equal to the arterial and capillary transit time,^27^ and high singleTI ASL signal is therefore not typically observed in the venous vasculature. However, if transit times are faster, labelled blood water may traverse the microcirculation without exchanging with tissue water.^25^ Such shunting may manifest as venous hyperintensities on CBF maps, which have also been observed in populations with arteriovenous fistulas and malformations, who lack capillary beds.^28–30^ In one recent singleTI ASL study, higher venous hyperintensity scores, assessed visually, were associated with reduced OEF in patients with SCA and SCI, but not in those without lesions.^26^ Although SCI were assessed using a low-resolution sequence, these findings indicate that hyperemic flow may lead to regionally inefficient OEF secondary to arteriovenous shunting in some patients with SCA.

Potential arteriovenous shunting could be further investigated using multiTI ASL techniques. MultiTI ASL involves acquiring images at a range of TIs, allowing the passage of labelled blood water to be fully quantified on a voxel-wise basis.^27^ These techniques thus enable the full hemodynamic signal to be captured, as opposed to just a snapshot at a single TI. This in turn eliminates the need for assumptions about transit times, providing CBF estimates that in theory are not sensitive to arterial transit time effects. There have, however, been relatively few studies in clinical populations. In the only two multiTI studies in patients with SCA to date, SCA was associated with increased CBF and reduced bolus (*i.e*. blood) arrival times (BAT) across the whole brain^31^ and in anterior, middle, and posterior cerebral artery territories, consistent with macrocirculatory hyperperfusion leading to faster transit times.^32^ However, these studies only examined hemodynamic parameters in gray matter. Further, no ASL study (cross-sectional or longitudinal) has attempted to quantify CBF or BAT from venous signal, or assess associations with cognition in an SCA population.

Therefore, this study used singleTI and multiTI ASL to estimate CBF and BAT in gray matter, white matter, and high-signal sagittal sinus regions in patients with SCA and controls. Based on the prior singleTI^6,7,25,26^ and multiTI ASL^32^ studies discussed above, it was hypothesised that across all regions, CBF would be increased irrespective of sequence, and BAT reduced in patients with SCA. Associations with arterial oxygen content (CaO_2_), disease-modifying treatments, SCI, and cognitive performance were also explored.

## Materials and methods

### Standard protocol approvals, registrations, and patient consents

West London NHS (SAC; 05/Q0408/42, 11/EM/0084, 15/LO/0347), Yorkshire NHS (POMS; 15/YH/0213), and University College London (14475/001) ethics committees provided approval, and participants/parents/legal guardians provided written informed consent according to the Helsinki Declaration of 1975.

### Participants

This retrospective cross-sectional study collected data from patients recruited to two concurrent studies with overlapping MRI and cognitive assessment protocols between 2015 and 2019: the Sleep Asthma Cohort follow-up (SAC)^33^ and the Prevention of Morbidity in Sickle Cell Anemia baseline investigation (POMS).^34^ Controls were siblings and race-matched peers (i.e. Black British) of patients recruited to SAC. Patients were ineligible for SAC and POMS study participation if they were receiving nocturnal respiratory support at the time of enrollment, participating in a clinical trial evaluating blood transfusion or oxygen therapy, or had chronic lung disease (other than asthma) or existing respiratory failure. Additional exclusion criteria for the POMS study were hospital admissions for acute sickle complications within 1 month of enrollment, more than 6 hospital admissions for acute sickle complications within 12 months of enrollment, overnight oximetry showing mean overnight saturation of less than 90% for more than 30% of total sleep time, severe sleep apnea defined by 4% oxygen desaturation index >15/h, and chronic blood transfusion or transfusion within 3 months of enrollment. For the SAC study, patients were enrolled without regard to past sickle- or sleep-related morbidity or transfusion status.

### Cognitive and socio-economic measures

IQ was estimated using the two-subtest Wechsler Abbreviated Scale of Intelligence (WASI; POMS participants),^35^ the Wechsler Intelligence Scale for Children (WISC-IV; SAC participants <16 years),^36^ or the Wechsler Adult Intelligence Scale (WAIS-IV; SAC participants >16 years).^36^ Subtests from the WISC-IV or WAIS-IV measuring working memory and processing speed were used to calculate composite indices (WMI and PSI, respectively). Executive function was assessed using the achievement score and completion time on the Tower test from the Delis-Kaplan Executive Function System (D-KEFS).^37^ Participants were assessed as close to the date of MRI as possible, with 76% undergoing both on the same day, and all undergoing both within 4.5 months.

### Socioeconomic measures

Education deciles, estimated from UK postcode using the English Indices of Deprivation,^38^ provided an index of socio-economic status. This measure has been associated with cognitive performance in SCA patients,^39^ and reflects educational attainment in local areas based on several indicators: (a) average scores for pupils in state-funded schools at ages 7–11 and 14–16 years, (b) absence from state-funded secondary schools, (c) the proportion of people staying on in education/training post 16 years, entry to higher education, and (d) proportion of working adults with no/low qualifications and language proficiency. Total scores are ranked from 1 to 10, with 1 representing the most deprived.

### Hematological measures and treatment

In patients, hydroxycarbamide/hydroxyurea use, chronic transfusion regimens, and the closest routine full blood count to date of MRI were collected from relevant medical records. Blood draws were considered unethical by the ethics board in controls. Hematocrit and hemoglobin were therefore estimated based on age and sex using literature values in this group.^40^ Peripheral oxygen saturation (SpO_2_) was recorded using a pulse oximeter. For four patients with missing hemoglobin, and seven controls with missing SpO_2_, group means were substituted. Assuming pO_2_, the partial pressure of oxygen, is 100 Torr in room air, CaO_2_ was calculated as;

$$\left(\text{1}.\text{34}\times \text{Hemoglobin }\left(\text{g}/\text{dl}\right)\times \%\text{Oxygen Saturation}\right)+\left(0.00\text{3}\times {\text{pO}}_{\text{2}}\right)$$

### MRI acquisition

MRI was performed on a 3 T Siemens Prisma (Erlangen, Germany) with 80 mT/m gradients and a 64-channel receive head coil at Great Ormond Street Hospital. The protocol included a prototype singleTI pseudo-continuous ASL (pCASL) acquisition, with background suppression, and a 3D gradient-and-spin-echo (GRASE) readout (repetition time [TR] = 4620 ms, echo time [TE]=21.8 ms, labeling duration = 1800 ms, post-labeling delay = 1500 ms, repetitions = 10, field of view = 220 mm, matrix size = 64 × 62, in-plane resolution = 1.7 × 1.7 mm [after zero-filling], number of partitions = 24, slice thickness = 4.0 mm, turbo factor = 12, echo-planar imaging [EPI] factor = 31, segments = 2, with parallel imaging, generalized autocalibrating partial parallel acquisition [GRAPPA] acceleration factor = 2). A proton-density weighted (M0) image was also acquired (TR = 4000 ms), with specifications identical to the pCASL acquisition but with the labeling pulses removed. Total acquisition time was 3 minutes 19 seconds. A prototype multiTI pulsed ASL (PASL) acquisition was also used, with background suppression, and a 3D GRASE readout (10 inflow times with one acquisition per TI, ranging 350–2600 ms in 250 ms intervals, and TR of 3300 ms; all other readout parameters were identical to the pCASL acquisition. Q2TIPS^41^ RF pulses were applied 700 ms after the labeling pulse to define the temporal width of the bolus. As for the singleTI acquisition, a proton-density weighted map was acquired (TR = 4000 ms) with labeling removed. The total acquisition time was 2 minutes 25 seconds. Other sequences included coronal high-resolution 3D FLAIR (TR = 5000 ms, TE = 395 ms, voxel size = 0.65 × 0.65 × 1.0 mm, scan time = 6 min 22 sec), axial 2D T2-w turbo spin echo (TR = 8420 ms, TE = 68 ms, voxel size = 0.50 × 0.50 ×4.0 mm, scan time = 2 min, 50 sec), T1-w magnetization-prepared rapid acquisition gradient echo (MPRAGE; TR = 2300 ms, TE = 2.74 ms, TI = 909 ms, flip angle = 8°, voxel size = 1 × 1 × 1 mm, scan time = 5 min, 21 sec), and 3D time-of-flight MRA (TR = 21.0 ms, TE = 3.4 ms, scan time = 5min, 33 sec).

### Radiological measures

The silent infarct transfusion trial (SIT) definition^42^ of SCI was used: a neuroradiologist (D.S) diagnosed SCI as regions of abnormally high signal intensity, consistent with ischemia, visible in two planes on FLAIR and T2-weighted MRI, measuring at least 3 mm in greatest dimension. Clearly distinguishable SCI mimics (*e.g.* perivascular spaces) were excluded. SCI burden was quantified using intensity thresholds as described previously.^43^

### Hemodynamic measures

Raw singleTI and multiTI ASL images were inspected, and excluded if there were major motion-related artifacts. As ASL model fitting requires a value representing the T1 relaxation rate of blood (T1_bl_), and T1_bl_ is dependent on hematocrit and SpO_2_, estimated values were calculated based on measured (patients) or estimated (controls) hematocrit and measured SpO_2_.^44^

In-house MATLAB scripts were used to pre-process the raw ASL images. SingleTI images were co-registered using the FSL *flirt* tool (version 6.0; FMRIB, Oxford, UK; https://fsl.fmrib.ox.ac.uk/), with affine registrations (12 degrees of freedom) derived from a correlation ratio cost function. The mean singleTI pCASL difference signal was calculated over the 10 co-registered repetitions, and used to estimate CBF following the method described in Alsop et al,^45^ with λ = 0.9, α = 0.85, τ = 1.8, and patient-specific estimates of T1_bl_ (mean SCA = 1.99, sd SCA = 0.09, mean control = 1.77, sd control = 0.07).

For the multiTI images, where motion occurred at later TIs (>1.35 s), images were co-registered using the FSL flirt tool, with affine registrations as described for the singleTI images. Where motion occurred at earlier inflow times with lower signal-to-noise ratio, datasets were excluded. To remove voxels with unreliable signal, four square ghost-free background ROIs with a width of 5 voxels were created and placed in each corner of the difference image timeseries. The mean and standard deviation of ghost-free background were computed. Reliable voxels were defined as those where the difference signal was at least greater than the mean + one standard deviation of ghost-free background on at least one image in the timeseries.^46^ The PASL difference signal was used to fit voxel-wise values of CBF and BAT using the general kinetic model described in Buxton,^27^ with λ = 0.9, α = 0.98, and τ = 0.7, T1_tissue_ = 1.9) and patient-specific estimates of T1_bl_ (values as for singleTI above). As we were interested in imaging the sagittal sinus, the T1 of tissue value used represented the average between the patient and control estimates of T1 blood.

### Regions of interest (ROIs)

**Figure 1.**
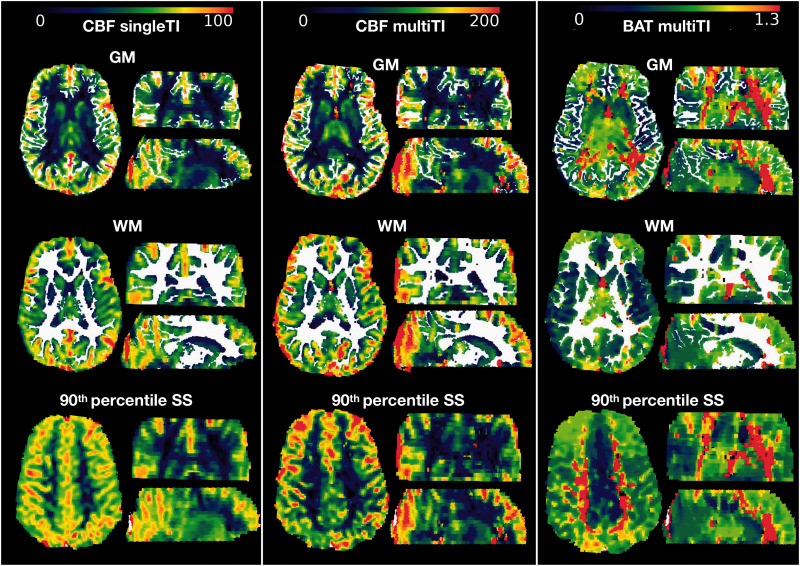
Regions of interest. Showing the gray matter (GM), white matter (WM), and high-signal sagittal sinus (90th percentile SS) regions of interest in each imaging plane overlaid in white on a single inflow time (singleTI) and multi inflow time (muliTI) cerebral blood flow (CBF) map and a multiTI bolus arrival time (BAT) map from a representative participant with sickle cell anemia (male, 8 years of age).

The mean of the unlabeled singleTI images, and the unlabeled multiTI image with the longest TI, were aligned with T1w images in native space using ANTs rigid and deformable transforms (i.e. antsRegistrationSyn; https://github.com/ANTsX/ANTs) with default settings. To create the gray and white matter ROI, cortical reconstruction and volumetric segmentation were performed on T1-w images using Freesurfer (Center for Biomedical Imaging, Massachusetts, USA; http://surfer.nmr.mgh.harvard.edu/). To correct for potential partial volume effects, gray and white matter masks were eroded using a kernel box with a width of 2 voxels in FSL. To create the high-signal sagittal sinus ROI, a publicly available vein atlas in Montreal Neurological Institute (MNI) standard space was used.^47^ From the atlas, a mask of the sagittal sinus was registered to each participant’s native T1-w image using the non-linear MNI transformation matrix from FSL’s *fsl_anat* pipeline. As for the gray and white matter ROIs, the registered sagittal sinus mask was eroded using a kernel box with a width of 2 voxels in FSL. As our ASL sequences did not provide whole-brain coverage, and coverage of the sagittal sinus ROI varied from 41%–97% (median = 69%) for the single TI sequence, regions with the highest signal were extracted; a lower threshold corresponding to the 90th percentile of voxels within the sagittal sinus mask was applied to each participant’s unmasked registered singleTI CBF map. This thresholding restricted analyses to the regions with the highest signal, creating the high-signal sagittal sinus ROI (Figure 1).

### Statistical analysis

Analyses were performed in RStudio Desktop 1.3.959 (http://www.rstudio.com/). Mean CBF and BAT values were extracted across ROIs for both sequences and, after assessing normality (Shapiro-Wilk test), compared between patients with SCA and controls using Student’s t-tests for normally, and Mann-Whitney U tests for non-normally, distributed variables on a region-by-region basis. Relationships between mean estimated CBF and BAT values were explored across ROIs using Pearson’s and Spearman’s Rho correlation coefficients. For the multiTI images, kinetic curves were also presented graphically.

For further exploration of hemodynamic variables using parametric analyses, non-normally distributed variables were log-transformed. Student’s t-tests were performed to compare age- and sex- adjusted mean estimated CBF and BAT across ROIs as a function of chronic transfusion status, hydroxycarbamide/hydroxyurea prescription, and presence of SCI. Pearson correlations were explored between age- and sex- adjusted hemodynamic parameters and SCI burden, CaO_2_ and cognitive performance.

Cognitive variables which showed a p-value of <0.1 in univariate analyses prior to correction for multiple comparisons were explored in multivariable linear regression models in each group, with pre-selected covariates comprising age, sex, hydroxycarbamide use, chronic transfusion status, SCI burden, total intracranial volume, and education-deciles.^39^ Model assumptions were assessed using the global validation of linear models package.^48^ Across analyses and for predictors in models, p < 0.05 was considered statistically significant. Multiple comparisons were corrected for using the Benjamini and Hochberg false discovery rate (FDR) procedure^49^ within groups (i.e. patients, controls) and across parameter maps (i.e. singleTI CBF, multiTI CBF, and multiTI BAT). This study did not assess ASL measures against the current clinical gold standard (i.e. PET), which is invasive. Estimated CBF and BAT values are therefore relative.

### Data availability statement

Fully anonymised data will be shared upon request from qualified investigators.

## Results

### Participants

Of 172 recruited participants, 94 patients (aged 8–27 years, 46 male, 93 HbSS, 1 HbSb_0_-thalassemia) and 42 controls (age 8-30 years, 16 male, 26 siblings, 23 HbAA, 17 HbAS, 2 HbAC) had useable singleTI and/or multiTI ASL data (Figure e1). Of the included participants, 3 patients had no cognitive data, 5 patients and 1 control had no D-KEFS data, 7 patients received regular transfusion, and 34 were prescribed hydroxycarbamide.

SCI were detected in 39 patients and 4 controls, while 2 patients had large vessel vasculopathy (1 right internal carotid narrowing, 1 right middle cerebral artery stenosis). CaO_2_ was lower in patients with SCA compared to estimated values in controls, but there were no differences between patients and controls in age, sex, or education deciles (Table 1).

**Table 1.** Sample demographics and cognitive performance.

	SCA (n = 94)	Control (n = 42)	Between-group differences
Demographic variables	Count (percentage)/Median (IQR)		
Sex	46 Male (48.94%)	16 Male (38.10%)	p = 0.32
Age (yr)	16.67 (13.32–19.89)	17.33 (14.57–20.48)	p = 0.65
Education Decile	5 (4 –7)	5 (4–6)	p = 0.72
Hematological variables			
Arterial oxygen content (CaO_2,_ mL/d)	11.79 (10.39–12.92)	17.69 (17.42–18.22)	p < 0.0001***
Radiological variables			
Silent Cerebral Infarction (SCI)	39 (41.49%)	4 (9.52%)	p = 0.0005***
SCI burden (1 mm^3^ voxels)	59 (33.5–149.5)	19 (15.0–35.5)	p = 0.11
Cognitive variables	Mean (SD)/Median (IQR)		
Intelligence quotient (IQ)	92.63 (13.44)	98.10 (12.00)	p = 0.02*
Working memory index (WMI)	91.73 (13.69)	99.24 (13.48)	p = 0.004**
Processing speed index (PSI)	89.49 (12.96)	97.55 (13.17)	p = 0.001***
Tower Completion Time	559.44 (147.44)	561.17 (151.88)	p = 0.95
Tower Achievement	9 (8–10)	9 (8–11)	p = 0.56
Hemodynamic variables	Mean (SD)/Median (IQR)		
sTI ASL	n = 89	n = 42	
Grey Matter CBF (ml/100 g/min)	54.06 (7.78)	42.52 (7.13)	p < 0.0001***
White Matter CBF (ml/100 g/min)	31.25 (4.82)	22.91 (4.49)	p < 0.0001***
90th percentile Sag. Sinus CBF (ml/100 g/min)	104.66 (80.06–145.60)	66.18 (57.03–78.93)	p < 0.0001***
mTI ASL	n = 80	n = 36	
Grey Matter CBF (ml/100 g/min)	129.02 (22.26)	89.62 (11.83)	p < 0.0001***
White Matter CBF (ml/100 g/min)	69.52 (11.99)	51.83 (7.46)	p < 0.0001***
90th percentile Sag. Sinus CBF (ml/100g/min)	253.77 (157.73–467.67)	145.83 (113.95–202.52)	
Grey Matter BAT (s)	0.72 (0.66–0.75)	0.82 (0.77–0.89)	p < 0.0001***
White Matter BAT (s)	0.96 (0.91–1.05)	1.15 (1.08–1.26)	p < 0.0001***
90th percentile Sag. Sinus BAT (s)	1.17 (0.98–1.45)	1.01 (0.87–1.10)	p = 0.0025**

Values are summary and test statistics. SCA ; sickle cell anemia; sTI; single inflow time sequence: mTI; multi inflow time sequence: CBF; cerebral blood flow: BAT; bolus arrival time: *90th%ile Sag. Sinus*; high-signal regions of sagittal sinus: SD; standard deviation: IQR; interquartile range: p;probability values for between-group differences. Full statistics are presented in the extended supplementary table (Table e1). ^p<0.1, *p<0.05, **p<0.01, ***p<0.001.

Cognitive performance was reduced in patients compared to controls, with average Wechsler scores 5 IQ points, 7 WMI points and 8 PSI points lower than controls (all *p* < 0.05; Table 1). There were no significant differences in executive function between groups, with similar D-KEFS Tower scores and completion times (Table 1). Cognitive performance in controls did not significantly differ as a function of sickle cell trait (all *p* > 0.05).

### Hemodynamic parameters

**Figure 2.**
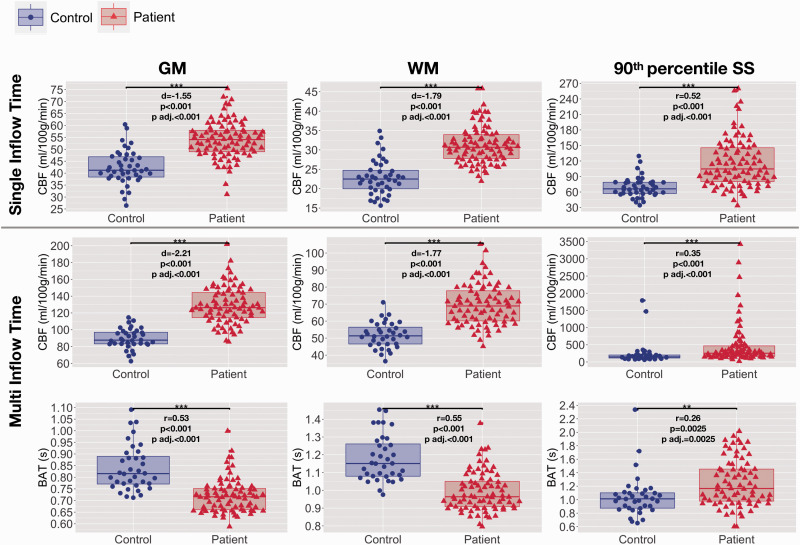
Hemodynamic parameters across regions of interest. Boxplots showing mean cerebral blood flow (CBF) and bolus arrival times (BAT) based on the single- and multi- inflow time sequences (rows) across different regions of interest (ROIs; columns) in patients with sickle cell anemia (shown in red) and healthy controls (shown in blue). Standardised mean differences (d) and probability values from independent t-tests (p) adjusted within parameter types for multiple comparisons using the Benjamini and Hochberg false discovery rate (p adj.) are displayed. GM: gray matter; WM: white matter 90th percentile; SS: high-signal sagittal sinus regions. ^p<0.1, *p<0.05, **p<0.01, ***p<0.001.

In both groups, visual inspection of the raw kinetic curves indicated relatively robust multiTI ASL signal across ROIs (Figure e2). Mean CBF was significantly higher in patients with SCA compared to controls across gray matter, white matter, and high-signal sagittal sinus regions, irrespective of sequence (all *p* < 0.05; Table 1, Figure 2). Mean BAT was also significantly shorter in patients with SCA compared to controls across gray and white matter (*p* < 0.0001; Table 1, Figure 2). However, across high-signal sagittal sinus regions, BAT was longer and there was greater variability in patients (*p* = 0.002; Table 1, Figure 2). All comparisons remained significant in the same direction when the two patients with large vessel vasculopathy were removed from the sample. There were no significant differences in CBF or BAT between controls with and without sickle cell trait (all *p* > 0.05).

**Figure 3.**
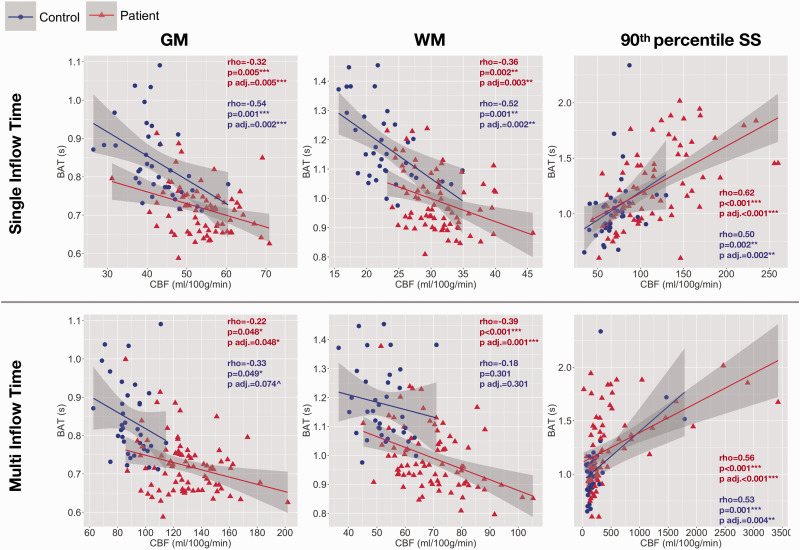
Associations between CBF and BAT. Scatterplots showing the relationship between cerebral blood flow based on the single and multi inflow time sequences and bolus arrival time across regions of interest in patients (shown in red) and controls (shown in blue). Values are Spearman’s correlation coefficients (rho) and p-values (p) adjusted within parameter types for multiple comparisons using the Benjamini and Hochberg false discovery rate (p adj.). GM: gray matter; WM: white matter; 90th percentile SS: high-signal sagittal sinus regions. ^p<0.1, *p<0.05, **p<0.01, ***p<0.001.

In patients, using both sequences, mean CBF and BAT were significantly negatively correlated across gray and white matter, but significantly positively correlated across high-signal sagittal sinus regions (all p < 0.05; Figure 3). In controls, the pattern was similar, but relationships were not significant across gray or white matter based on the multiTI sequence (p > 0.05, Figure 3).

### Associations with CaO_2_

**Figure 4.**
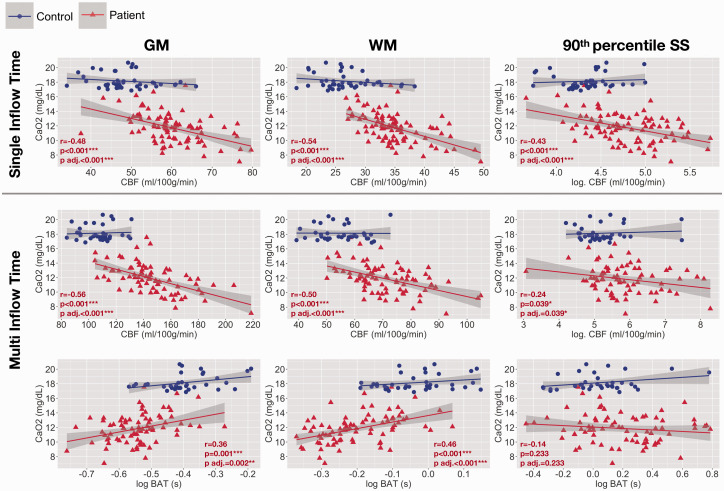
Associations with arterial oxygen content. Scatterplots showing the relationship between arterial oxygen content and age- and sex- adjusted mean cerebral blood flow (CBF) and bolus arrival time (BAT) based on the single- and multi- inflow time sequences (rows) across different regions of interest (columns) in patients with sickle cell anemia (shown in red). Pearson’s correlation coefficients (r) and p-values (p) adjusted within parameter types for multiple comparisons using the Benjamini and Hochberg false discovery rate (p adj.) are displayed. GM: gray matter; WM: white matter; 90th percentile SS: high-signal sagittal sinus regions; log: log transformed. ^p<0.1, *p<0.05, **p<0.01, ***p<0.001.

In patients with SCA, age- and sex- adjusted mean CBF was negatively correlated with CaO_2_ across gray matter, white matter, and high-signal sagittal sinus regions, irrespective of whether the singleTI or multiTI sequence was used (all *p* < 0.05, Figure 4). Age- and sex- adjusted mean BAT was positively correlated with CaO_2_ across gray and white matter (both *p* = <0.05), but not across high-signal sagittal sinus regions (*p* = 0.30, Figure 4).

### Associations with treatment

In patients with SCA, there were no significant differences in hemodynamic parameters as a function of hydroxycarbamide prescription (Figure e3). However, age- and sex- adjusted mean CBF was significantly reduced across gray and white matter in patients receiving monthly transfusions compared to those not receiving monthly transfusions using the singleTI sequence (both *p* < 0.05, Figure e4). Using the multiTI sequence, the effects did not reach significance (both *p* > 0.05). No further effects of disease modifying treatments were observed (Figure e4).

### Associations with SCI

There were no effects of presence of SCI on age- and sex- adjusted CBF or BAT across any ROI in patients with SCA (all *p* > 0.05, Figure e5). However, in patients with SCI, using the multiTI sequence, there was a significant positive correlation between age- and sex- adjusted CBF and SCI burden across white matter (*p* = 0.03, Figure e6).

**Figure 5.**
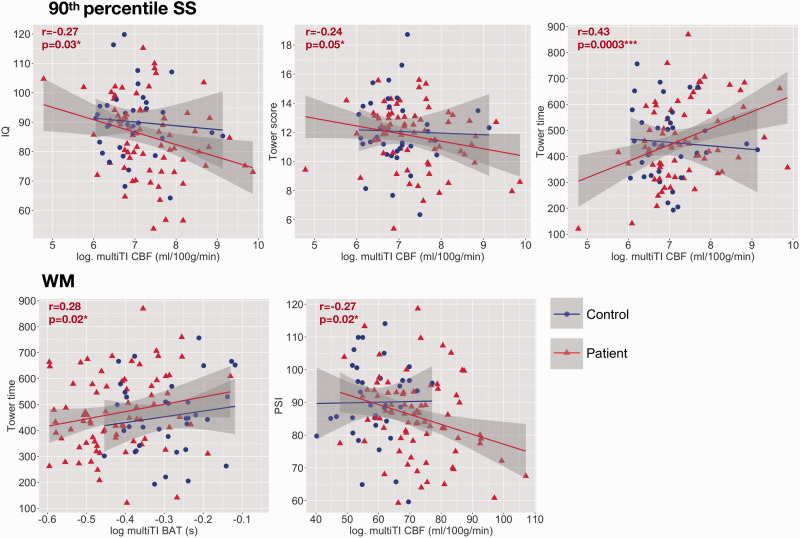
Associations with cognitive performance. Scatterplots showing relationships between cognitive performance and mean cerebral blood flow (CBF) and bolus arrival times (BAT) across high-signal sagittal sinus regions (90th percentile SS: top) and white matter (WM: bottom) based on the multi inflow time sequence (multiTI) in patients with sickle cell anemia (shown in red). Values are Pearson’s (r) partial correlation coefficients and p-values (p) from the regression models. Variables are adjusted for other variables in the model; age, male sex, chronic transfusion, hydroxycarbamide, SCI burden, total intracranial volume, and education deciles. ^p<0.1, *p<0.05, **p<0.01, ***p<0.001.

### Associations with cognition

In patients, multiple linear regression analyses found that lower IQ and Tower scores and longer Tower completion times were predicted by higher CBF based on the multiTI sequence across high-signal sagittal sinus regions, and that lower PSI was predicted by higher CBF based on the multiTI sequence across white matter (all p < 0.05; Table 2, Figure 5). Tower completion times were additionally predicted by increases in BAT across white matter (p = 0.03, Table 2, Figure 5). No such associations with cognitive performance were observed in controls, or for any other hemodynamic parameter.

**Table 2.** Regression models.

		Outcome: IQ					Outcome: Tower achievement score				
Predictors	b	β	95% CI	p	r	Predictors	b	β	95% CI	p	r
Log. multiTI CBF 90th percentile Sag. Sinus	−3.94	−0.26	−7.41–−0.47	0.03*	−0.27	Log. multiTI CBF 90th percentile Sag. Sinus	−0.57	−0.23	−1.14–0.01	0.05*	−0.24
Age	−0.26	−0.09	−0.91–0.39	0.43	−0.10	Age	0.07	0.15	−0.04–0.18	0.20	0.16
Male Sex	−5.64	−0.21	−13.51–2.23	0.16	−0.17	Male Sex	−0.99	−0.22	−2.29–0.31	0.13	−0.19
Chronic Transfusion	9.09	0.17	−3.51–21.68	0.15	0.18	Chronic Transfusion	0.57	0.06	−1.50–2.64	0.58	0.07
Hydroxycarbamide	7.09	0.25	0.55–13.63	0.03*	0.26	Hydroxycarbamide	−0.57	−0.12	−1.65–0.51	0.29	−0.13
SCI burden	−0.004	−0.20	−0.009–0.001	0.096^	−0.20	SCI burden	−0.0001	−0.03	0.0007–−0.2496	0.80	−0.03
eTIV	0.004	0.04	−0.025–0.033	0.80	0.03	eTIV	−0.003	−0.19	−0.008–0.002	0.19	−0.16
Education Deciles	0.50	0.08	−1.00–2.00	0.51	0.07	Education Deciles	0.03	0.02	−0.22–0.27	0.83	0.03
		Outcome: Tower completion time					Outcome: PSI				
Log. multiTI CBF 90th percentile Sag. Sinus	70.44	0.41	33.385–107.507	0.0003***	0.43	multiTI CBF WM	−0.29	−0.27	−0.54–−0.04	0.02*	−0.27
Log. multiTI BAT WM	416.65	0.27	60.64–772.66	0.02*	0.28	−					
Age	0.13	0.004	−7.32–7.58	0.97	0.004	Age	−0.64	−0.23	−1.24–−0.04	0.04*	−0.25
Male Sex	26.28	0.09	−57.84–110.41	0.53	0.08	Male Sex	−9.77	−0.37	−17.00–−2.53	0.01**	−0.31
Chronic Transfusion	40.90	0.07	−93.09–174.89	0.54	0.08	Chronic Transfusion	−1.01	−0.02	−12.93–10.91	0.87	−0.02
Hydroxycarbamide	0.76	0.002	−69.19–70.70	0.98	0.003	Hydroxycarbamide	−3.81	−0.14	−9.71–2.10	0.20	−0.15
SCI burden	0.01	0.06	−0.04–0.07	0.61	0.06	SCI burden	0.0006	0.03	−0.004–0.005	0.81	0.03
eTIV	0.31	0.27	−0.005–0.62	0.05*	0.24	eTIV	0.01	0.14	−0.01–0.04	0.31	0.12
Education Deciles	−5.48	−0.07	−21.72–10.77	0.50	−0.08	Education Deciles	1.34	0.21	−0.04–2.72	0.06^	0.23

Values are unstandardised regression coefficients (b), standardised regression coefficients (**β**), probability values (p), partial correlation coefficients (r), and 95% confidence intervals (CI) from regression models in patients with sickle cell anemia (SCA). Log = log transform, multiTI= multi inflow time sequence, CBF=cerebral blood flow, BAT= bolus arrival time, 90th%ile Sag Sinus= high-signal sagittal sinus regions, WM=white matter, SCI=silent cerebral infarction, eTIV=estimated total intracranial volume. ^p<0.1, *p<0.05, **p<0.01, ***p<0.001.

## Discussion

Using singleTI and multiTI ASL, this study investigated CBF and BAT in patients with SCA and controls across gray matter, white matter, and high-signal sagittal sinus regions. Across gray and white matter, increases in CBF and reductions in BAT were observed in association with reduced CaO_2_ in patients with SCA, irrespective of sequence. Across high-signal sagittal sinus regions, CBF was also increased in association with reduced CaO_2_ using both sequences, but BAT was increased rather than reduced in patients across these regions, and there was no association with CaO_2_. Using the multiTI sequence in patients, increases in CBF across high-signal sagittal sinus regions were associated with reductions in IQ and Tower scores and longer Tower completion times, while increases in CBF and BAT across white matter were associated with reduced PSI and longer Tower completion times, respectively. Overall, the results indicate regional hemodynamic stress in patients with SCA with small, but significant, downstream effects on cognitive performance.

### Hemodynamic parameters

Across ROIs, estimates of CBF based on the multiTI sequence were higher than those based on the singleTI sequence in patients and controls. This was expected, given that the multiTI sequence captures the flow of labeled blood-water through the large arteries at early arrival times, which gives rise to high CBF values in voxels containing these arteries. Despite these differences, results based on both ASL sequences indicated higher CBF and shorter BAT across gray and white matter in association with reduced CaO_2_ in patients with SCA. These results support and extend those of prior studies^6,32^ and indicate that blood flows faster and arrives earlier across these regions in patients with SCA, potentially reflecting compensatory vasodilation to maintain brain tissue oxygenation despite reduced blood oxygenation. Using both sequences, for the first time quantitatively, we also demonstrated that patients with SCA exhibited increases in CBF in high-signal regions of the sagittal sinus. Two smaller-scale cross-sectional singleTI ASL studies had previously reported this but qualitatively,^25,26^ while a case report showed reduction in CBF across the sagittal sinus post-transfusion.^50^

For consistency with these prior studies, we defined high-signal sagittal sinus regions in individual patients on the basis of singleTI CBF maps. Interestingly, along with increased CBF, longer and more heterogeneous BAT was observed in patients with SCA across these regions. There was no relationship between BAT across high-signal sagittal sinus regions and CaO_2_, although the relationship persisted for CBF in these regions based on both sequences. Of note, in three patients, we observed extremely high values of around 2500 ml/100g/min using the multiTI sequence in these regions. The raw images and kinetic curves indicated that the effects in these patients were not artefactual, with the high signal at later inflow times likely explaining the high estimated values (Figure 2, Figure e7). As there are no prior quantitative data for comparison, further work, e.g. using PET, will be important to understand the heterogeneity observed.

The prior singleTI ASL studies describing high signal in the sagittal sinus qualitatively in patients with SCA,^25,26^ along with the recent single case-study,^50^ proposed that the effect may be due to hyperemia accelerating blood transit times through the microvasculature, with the faster flow causing labeled blood water to traverse the microvasculature without exchanging with tissue water. However, if this were the case, we would expect to see shorter rather than longer BAT in high-signal regions of the sagittal sinus in patients with SCA. Findings from our larger-scale quantitative study instead suggest that although CBF is higher in sagittal sinus regions, blood takes longer to get to there. One possible explanation for this pattern of results is that there is macro-circulatory hyperperfusion in the arterial tree, combined with altered capillary and/or venular flow patterns, as a result of increased micro-circulatory resistance secondary to abnormal erythrocyte rheology.^1,51^

Consistent with this notion, in other vascular beds, such as the cutaneous microcirculation of the forearm, prior studies have shown altered flow in patients with SCA, with unique periodic oscillations of high flow that are not observed in control populations.^52,53^ Authors have proposed that these periodic high-flow oscillations may serve to overcome obstruction and/or increased resistance in the microcirculation caused by the abnormal rheological properties of polymerized sickle hemoglobin, including increased rigidity and density.^54^ More work is required to establish whether a similar mechanism could explain the increased CBF and longer BAT observed in sagittal sinus regions in the current study. Determining whether altered capillary and/or venular micro-circulatory flow patterns reduce the efficiency of oxygen unloading in the brain, leading to hypoperfusion due to “functional/physiological shunting”,^55^ is also an important question for future research.

Clinically, future studies on how regional ASL measures relate to transcranial-doppler (TCD) measurements of cerebral blood flow velocity (CBFV) are likely to be informative. Although TCD is the gold standard screening measure for risk of stroke in children with SCA,^56^ the major limitation is that velocity increases may be due to narrowing of the blood vessel or increase in flow and/or turbulence within it.^1^ Further comparative studies with ASL, perhaps including the TCD resistance index^57^ and/or the pulsatility index which, in common with high-signal sagittal sinus CBF, is associated with IQ,^58^ may help refine TCD classifications by identifying the key hemodynamic factors associated with poor neurological and cognitive outcomes.

### Effects of disease-modifying treatments

Using a singleTI pCASL sequence, the case report mentioned above reported a 102.2% decrease in sagittal sinus CBF signal following an exchange transfusion in a 9-year old patient with SCA, which the authors interpreted as consistent with a reduction in shunting.^50^ Global OEF increases were also observed irrespective of calibration model.^50^ No change in global CBF was observed, further emphasising the need to explore hemodynamic parameters regionally in patients with SCA.

We also observed significantly reduced gray and white matter CBF using the singleTI sequence, in our case in chronically transfused patients with SCA. Although the effects did not reach significance using the multiTI sequence, this was likely due to reduced statistical power, with only 5 of the 7 chronically transfused patients having useable multiTI data and the pattern remaining the same in this subset (see Figure e4). Of note, we did not have data on age at first transfusion, and time between most recent transfusion and MRI was not controlled. Prior studies have established that the effects of transfusion on hemodynamic parameters and cognition may be relatively transient, reducing over time.^59,60^

We observed no significant differences in hemodynamic parameters as a function of hydroxycarbamide prescription. As for transfusion, age at first prescription and prescription duration were not controlled in our sample. There were also no available data on adherence. Whilst these findings point to possible normalization of some hemodynamic parameters with treatment in patients with SCA, future large-scale longitudinal studies are required, ideally with controlled timelines and pre- and post- treatment MRI.

### Associations with cognitive performance

Using the multiTI sequence, higher CBF across high-signal sagittal sinus regions was associated with lower IQ and executive function in patients with SCA, but not controls. Additionally, longer BAT across white matter was associated with slower performance on the Tower test, and higher CBF across white matter was associated with reduced PSI. Effect sizes were small to moderate. No associations between cognition and CBF were observed using the singleTI sequence, potentially underscoring the need for CBF estimations to take differences in BAT into account in these regions.

Overall, these findings support and extend those of prior studies reporting associations between elevated global TCD velocities^61^ and CBF^62^ and poorer executive functioning in patients with SCA. Importantly, our findings indicate possible regional differences in such associations, with associations observed for parameters across white matter and high-signal sagittal sinus regions, but not gray matter.

Taken together, the findings are also in line with those from a recent study where executive abilities were better soon after, as opposed to long after, an exchange transfusion,^60^ along with the aforementioned case study reporting a decrease in sagittal sinus CBF signal following exchange transfusion.^50^ Further work is, however, required to establish the mechanisms underlying improvements in higher-level cognitive abilities following a transfusion.

### Effects of SCI

In agreement with one prior qualitative study that observed no effects of SCI on venous hyperintensity score,^26^ we also found no associations between presence of SCI and CBF or BAT across gray matter, white matter, or high-signal sagittal sinus regions in our sample of patients with SCA. However, using the multiTI sequence in those with SCI, we observed a significant association between higher SCI burden and higher CBF across white matter. It is possible that this too reflects an association between poorer oxygen extraction efficacy related to increased flow and tissue ischemia. However, longitudinal studies are also required to better understand potential effects of chronic and acute changes in hemodynamic parameters on the development both of SCI and more widespread reductions in tissue integrity.^15,39,63^

### Limitations

For consistency with prior studies, this study defined high-signal sagittal sinus regions on singleTI CBF maps. An alternative approach would be to define these regions based on maps from the multiTI sequence, taking both BAT and CBF into account, with further study of the relationship between these parameters. Comparing approaches where signal is averaged along the entire sagittal sinus to the approach taken here, where smaller high-signal regions were extracted on an individual basis, would also be informative. It was not possible to optimize the entire sagittal sinus approach in this sample given the limited and variable field of view of the sequences, which covered the forebrain, with only partial coverage of the most superior cortical slices, and of superior regions of the midbrain. Whole brain coverage would require a longer echo train, with decreased signal-to-noise ratio (SNR), or more images, increasing scan times and motion susceptibility. Future studies should also explore the possibility of incorporating not only the arrival time and strength/magnitude of the venous outflow signal, but also the spatial distribution, which may improve the sensitivity to cognitive outcome.

One limitation inherent to ASL studies in patients with SCA is the potential for differences in labeling efficiency, which may affect CBF estimation.^31,64^ The primary contributors to altered labelling efficiency, increased velocities and B1^+^ under-excitation,^64^ were not assessed in this sample. Of note, only two patients with SCA in this sample had large vessel vasculopathy in the brain, and excluding them did not change the overall pattern of results. Although cervical stenosis, an additional potential contributor to differences in ASL labeling efficiency, cannot be excluded, it appears to be relatively less common in the absence of intracranial vasculopathy.^65^ ASL is also limited by possible differences in T1_blood_ in patients with SCA. Although we accounted for the effect of blood hematocrit on T1_blood_, methemoglobin measurement was not available in this sample and may also influence T1_blood_. Whilst direct measurement of venous T1_blood_ has been proposed,^66^ ASL measures are largely influenced by arterial T1_blood,_ and the relationship between venous and arterial T1_blood_ is unclear. Such measures were also not available in this sample.

A related limitation is that the T1_tissue_ value used in the Buxton model, which represented the average between the T1_blood_ of patients and controls, may be inaccurate – particularly for blood-water that may have left the blood pool, and re-entered the venous vasculature. However, it is impossible to determine this value, as blood-water may also have remained in the blood pool without exchanging with tissue. We chose this value because we were primarily interested in quantifying signal from the sagittal sinus (i.e. a blood pool) and considered that different fitting techniques for different tissue types would render the analysis overly complex and inconsistent. This was also why we elected not to employ a multi-compartment model or a scaling factor to reduce the effects of T1 relaxation, despite the evidence that these may be advantageous in gray matter.^23,24,45^ Although the T1_tissue_ value used is likely too high for the gray and white matter ROIs, this will not have affected the fitted BAT values, and would only have had a small effect on the fitted CBF values. Whilst the absolute fitted values in these regions should therefore be interpreted with caution, and were higher than those based on the singleTI sequence and prior multiTI ASL work in gray matter,^31,32^ the key comparisons were in the same direction and overall agreement. Importantly, the strength of correlations between CBF values and other parameters will not have been impacted.

A further consideration when using multiTI ASL is the optimization of inflow times. It is important to wait long enough for the labeled blood water to reach the regions of interest. In this study, the raw kinetic curves demonstrated robust signal across ROIs, including the sagittal sinus, where BAT was on average 1.01 s in controls, and 1.17 s in patients (Table 1); short enough that the inverted magnetization in labeled blood-water had not fully recovered due to T1 relaxation. Although robust, signal was substantially lower across white matter in both groups, which may have reduced the accuracy of estimates in this region. Further, despite the erosion of masks and removal of voxels with unreliable signal, it is possible that through-plane blurring and/or partial volume effects may have confounded signal across ROIs. The possibility that spill-over of gray matter signal accounts for the sagittal sinus signal observed in most controls and many patients, with the longer BAT and CBF observed driven by a subset in whom labelled arterial blood is actually detected, cannot therefore be excluded. However, the correlations between CBF and BAT are reversed in the sagittal sinus and not in gray and white matter in both patients and controls (Figure 3) which would not be expected if this effect represented spill-over of signal from other regions. Further, such large raw difference signal in both groups in this region (particularly given our noise removal step) would not be expected if the signal observed represented noise (Supl Figure e2).

Other limitations of this study include our lack of a gold-standard measure of CBF (e.g. PET), which meant that it was not possible to assess the accuracy of the ASL methods, as well as our lack of a direct measure of socio-economic status, which is known to influence cognitive performance.^67^ Blood draws were also considered unethical by the ethics board in controls, so we were not able to explore possible associations with CaO_2_ in this group. Strengths of this study include our large sample, novel quantification of venous signal, use of both singleTI and multiTI ASL, and inclusion of cognitive measures.

## Conclusion

Our findings are indicative of regional cerebral hemodynamic stress in patients with SCA, and are consistent with small but significant downstream effects on cognition. Future studies should utilise multiTI ASL to further investigate and optimize pipelines for biomarkers of regional hemodynamic stress and potential oxygen supply-demand mismatch, ideally using whole-brain sequences longitudinally and including patients on established and novel treatments. With further validation and optimization, such biomarkers may hold promise in providing objectively quantifiable and functionally significant endpoints for trials of novel treatments.

## Supplemental Material

sj-pdf-1-jcb-10.1177_0271678X211072391 - Supplemental material for Venous cerebral blood flow quantification and cognition in patients with sickle cell anemia Click here for additional data file. Supplemental material, sj-pdf-1-jcb-10.1177_0271678X211072391 for Venous cerebral blood flow quantification and cognition in patients with sickle cell anemia by Hanne Stotesbury, Patrick W Hales, Melanie Koelbel, Anna M Hood, Jamie M Kawadler, Dawn E Saunders, Sati Sahota, David C Rees, Olu Wilkey, Mark Layton, Maria Pelidis, Baba PD Inusa, Jo Howard, Subarna Chakravorty, Chris A Clark and Fenella J Kirkham in Journal of Cerebral Blood Flow & Metabolism
