# Supplementary material for: Venous cerebral blood flow quantification and cognition in patients with sickle cell anemia
Source: J Cereb Blood Flow Metab. 2022 Jan 6;42(6):1061–77. doi: 10.1177/0271678X211072391 (PMC9121533; doi:10.1177/0271678X211072391)
Supplement: sj-pdf-1-jcb-10.1177_0271678X211072391 - Supplemental material for Venous cerebral blood flow quantification and cognition in patients with sickle cell anemia [file sj-pdf-1-jcb-10.1177_0271678X211072391.pdf]

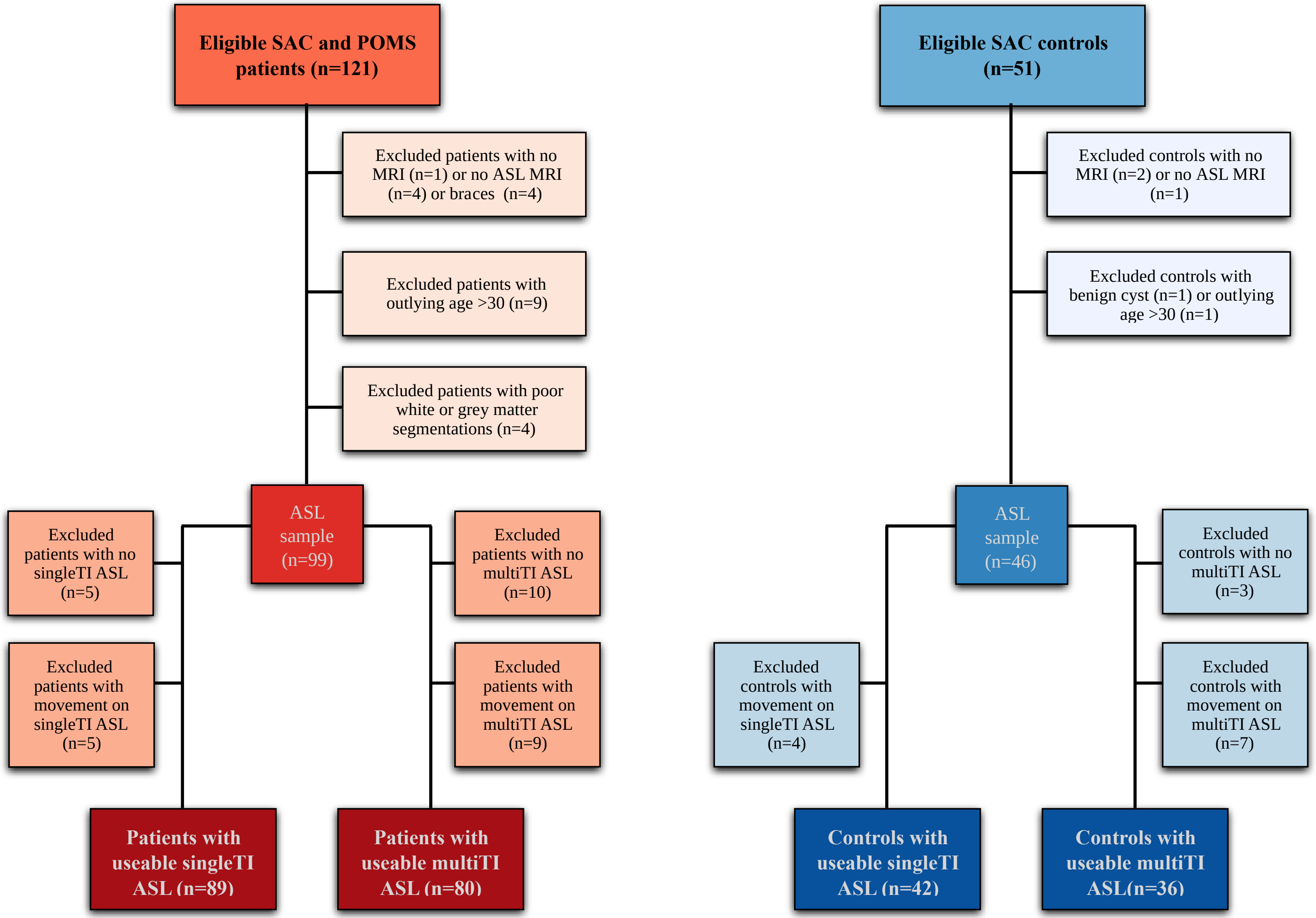

Figure e1. Participant flow-chart. Visualisation of reasons for participant exclusion

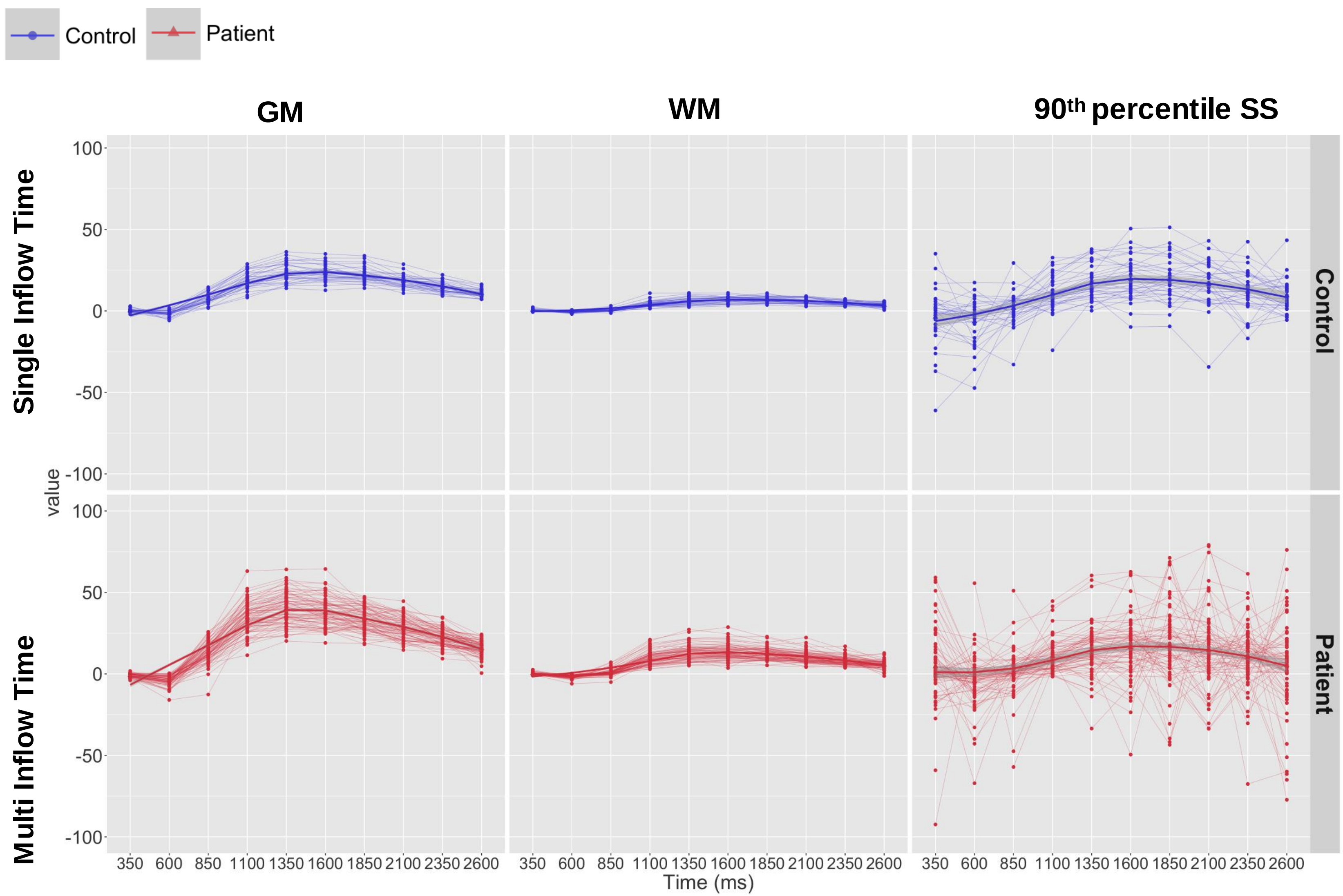

Figure e2. Kinetic curves. Line-plots showing raw kinetic curves (i.e. difference signal at each timepoint) from the multi inflow time sequence across regions of interest in patients (bottom: shown in red) and controls (top: shown in blue). GM= gray matter, WM= white matter, 90th %ile SS = high-signal sagittal sinus regions.

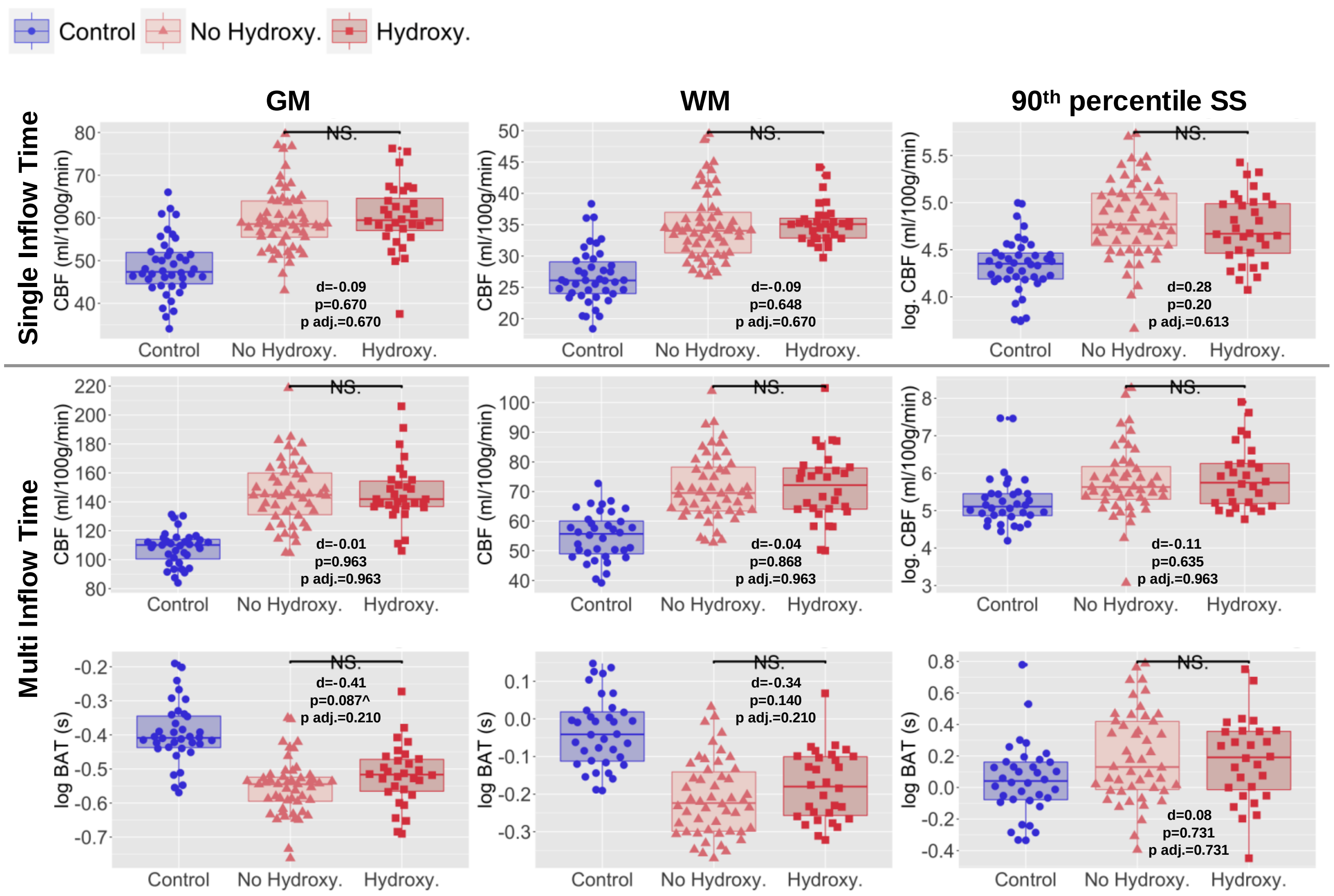

Figure e3. Hemodynamic parameters as a function of hydroxycarbamide. Boxplots showing age- and sex- adjusted mean cerebral blood flow (CBF) and bolus arrival times (BAT) based on the single- and multi- inflow time sequences (rows) across different regions of interest (ROIs; columns) in patients with sickle cell anemia (shown in red) and healthy controls (shown in blue) with (Hydroxy.) and without (No Hydroxy.) hydroxycarbamide prescription. Standardised mean differences (d) and probability values from independent t-tests (p) adjusted within parameter types for multiple comparisons using the Benjamini and Hochberg false discovery rate (p adj.) are displayed. GM=gray matter, WM=white matter, 90th%ile SS = high-signal sagittal sinus regions, log=log transformed.

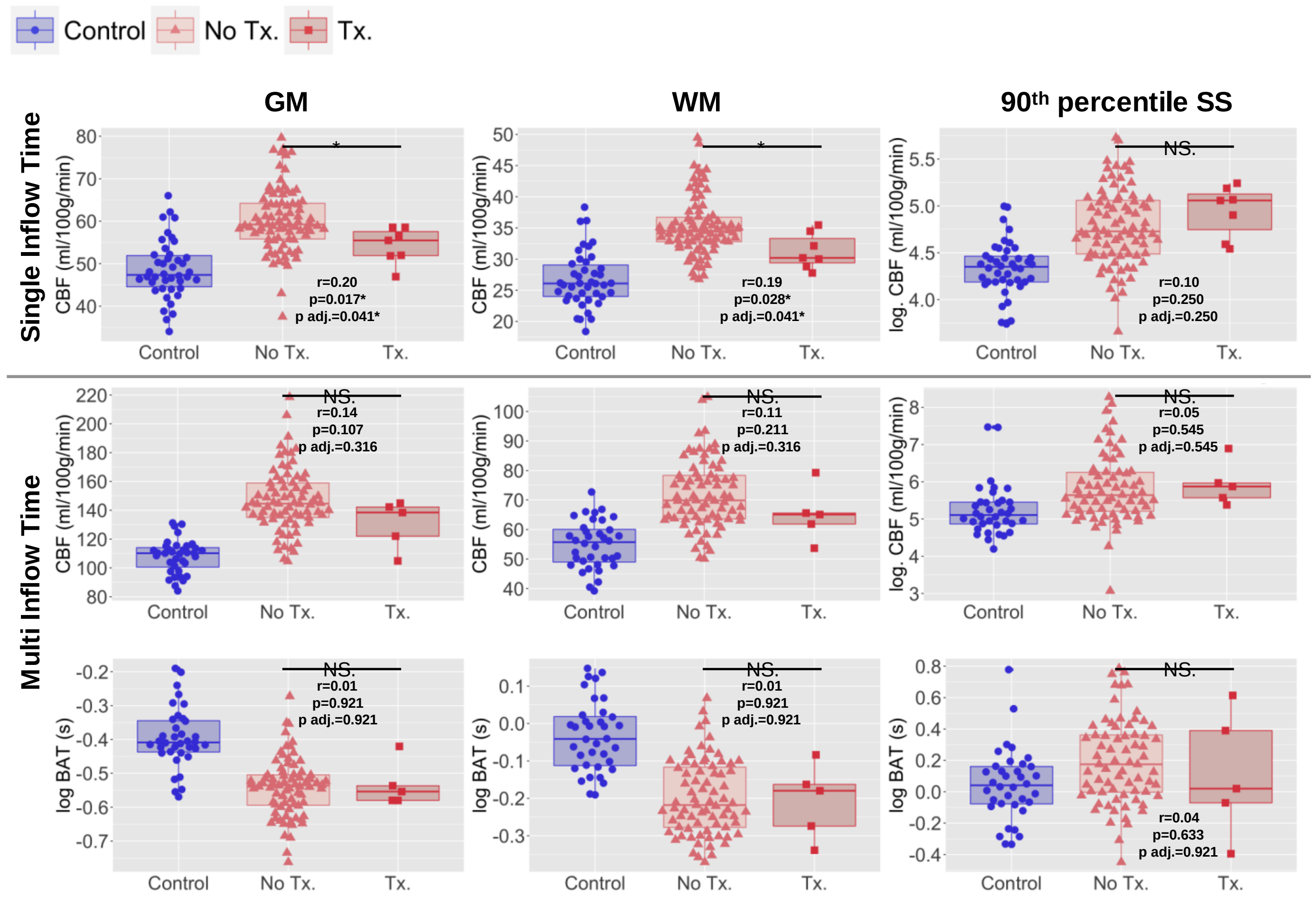

Figure e4. Hemodynamic parameters as a function of chronic transfusion. Boxplots showing age- and sex- adjusted mean cerebral blood flow (CBF) and bolus arrival times (BAT) based on the single- and multi- inflow time sequences (rows) across different regions of interest (ROIs; columns) in patients with sickle cell anemia (shown in red) and healthy controls (shown in blue) on (Tx.) and off (No Tx.) chronic transfusion regiments. Wilcoxon effect sizes (r) and probability values (p) adjusted within parameter types for multiple comparisons using the Benjamini and Hochberg false discovery rate (p adj.) are displayed. GM=gray matter, WM=white matter, 90th%ile SS = high-signal sagittal sinus regions, log=log transformed.

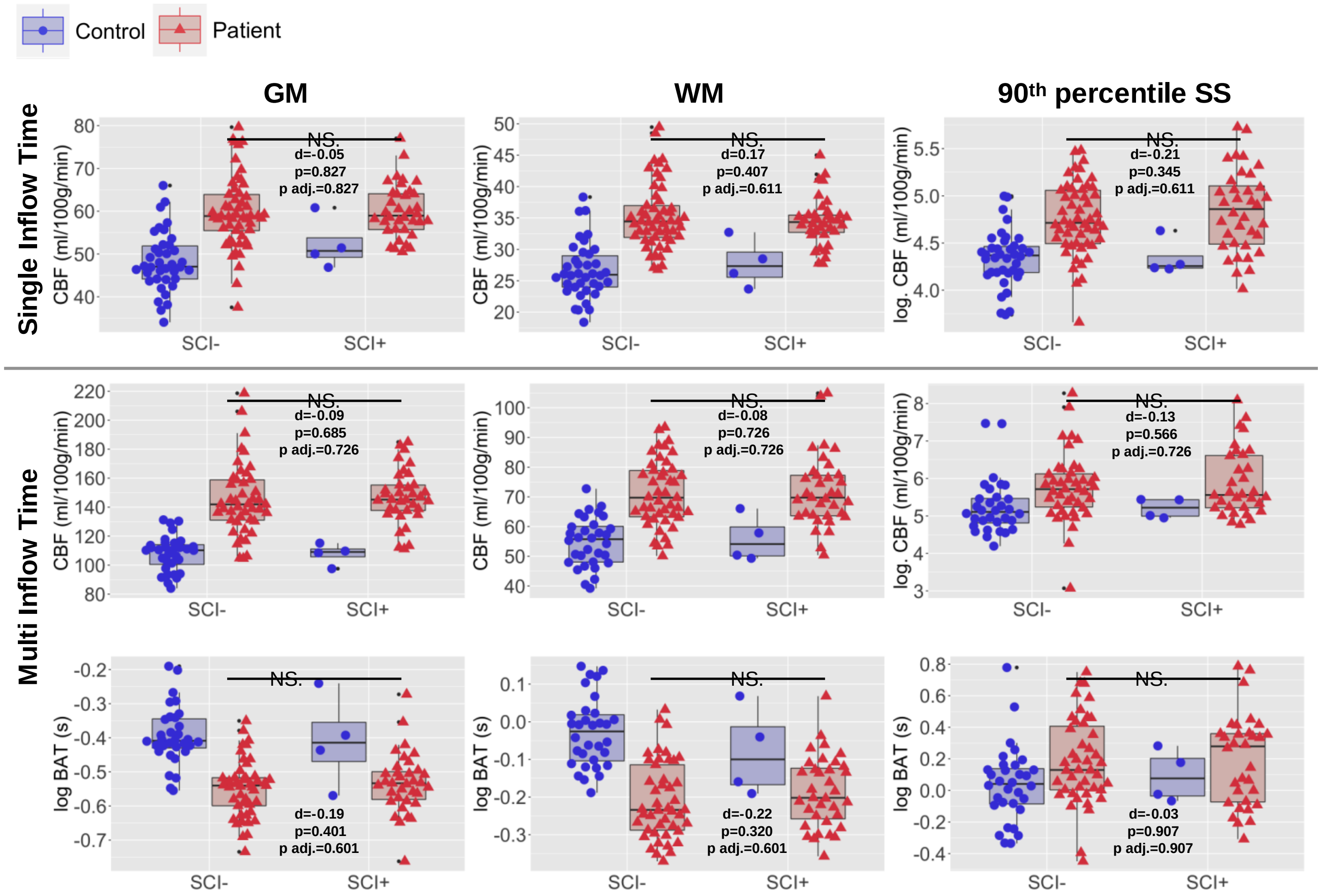

Figure e5. Hemodynamic parameters as a function of SCI. Boxplots showing age- and sex- adjusted mean cerebral blood flow (CBF) and bolus arrival times (BAT) based on the single- and multi- inflow time sequences (rows) across different regions of interest (ROIs; columns) in patients with sickle cell anemia (shown in red) and healthy controls (shown in blue) with (SCI+) and without (SCI-) silent cerebral infarction. Standardised mean differences (d) and probability values from independent t-tests (p) adjusted within parameter types for multiple comparisons using the Benjamini and Hochberg false discovery rate (p adj.) are displayed. GM=gray matter, WM=white matter, 90th%ile SS = high-signal sagittal sinus regions, log=log transformed.

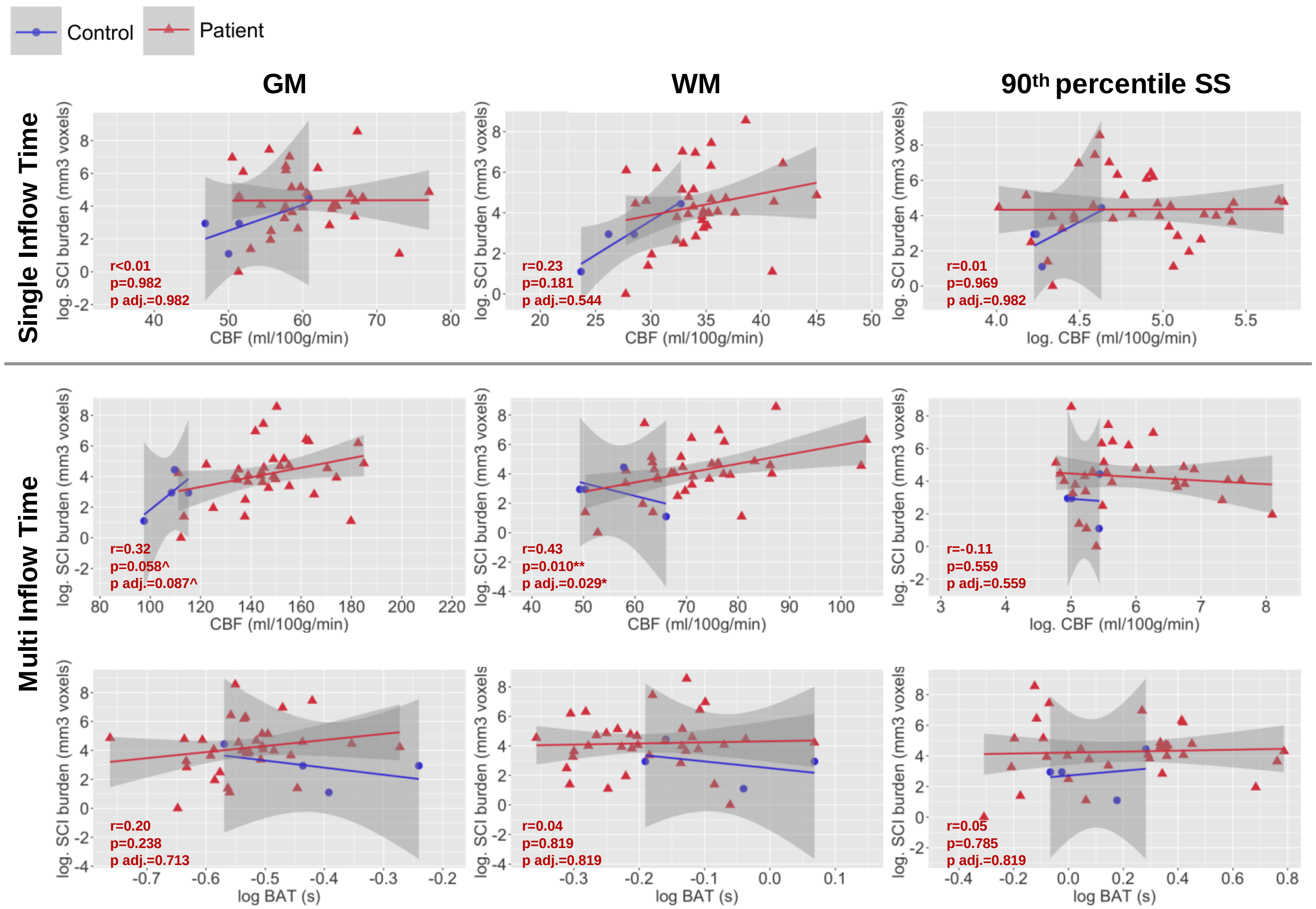

Figure e6. Associations with SCI burden. Scatterplots showing the relationship between silent cerebral infarct (SCI) burden and age- and sex-adjusted mean cerebral blood flow (CBF) and bolus arrival time (BAT) based on the single- and multi- inflow time sequences (rows) across different regions of interest (columns) in patients with sickle cell anemia (shown in red). Pearson's correlation coefficients (r) and p-values (p) adjusted within parameter types for multiple comparisons using the Benjamini and Hochberg false discovery rate (p adj.) are displayed. GM=gray matter, WM=white matter, 90th%ile SS = high-signal sagittal sinus regions, log=log transformed.

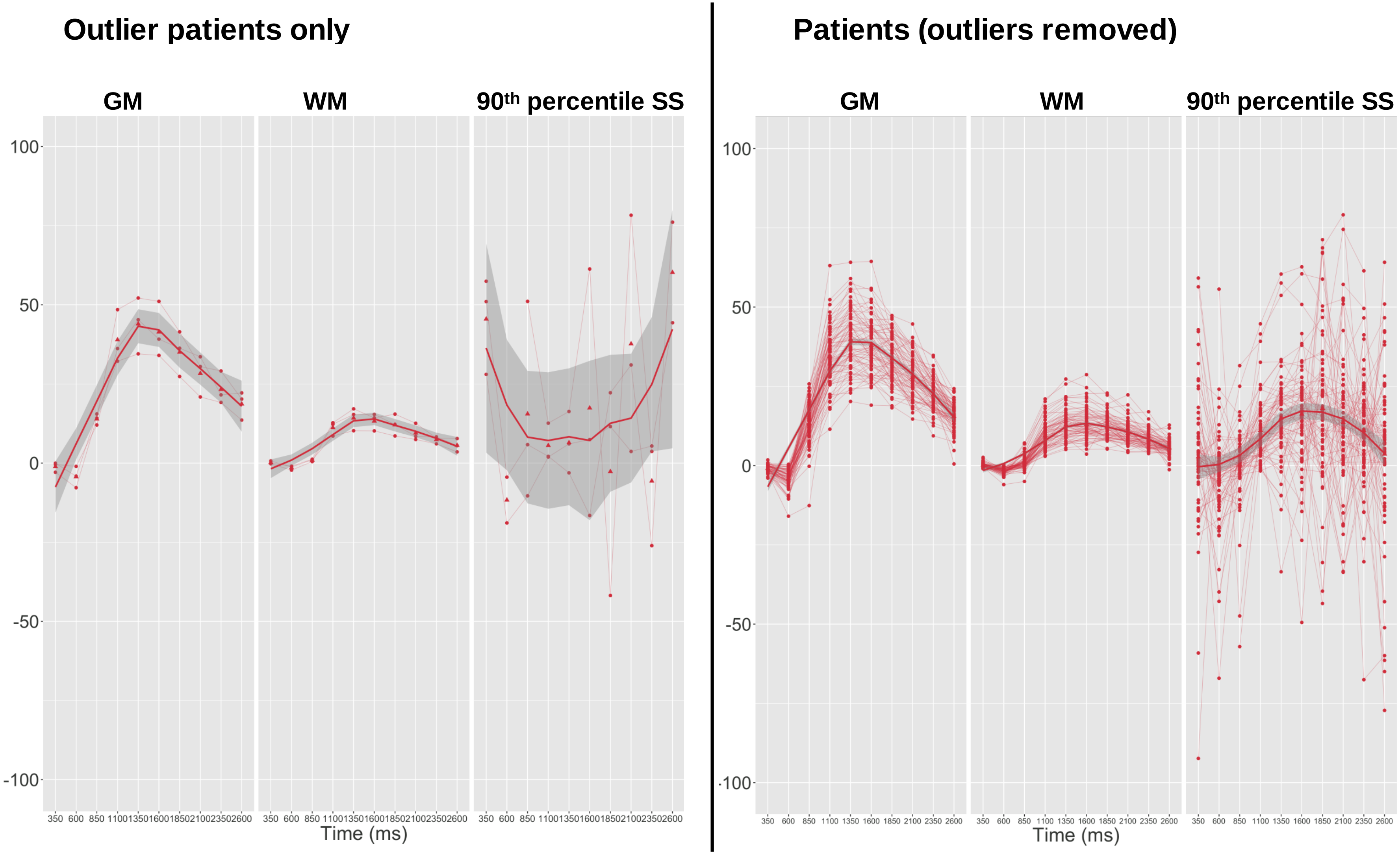

Figure e7. Kinetic curves in patients with outlying CBF. Line-plots showing raw kinetic curves (i.e. difference signal at each timepoint) from the multi inflow time sequence across regions of interest in three patients with very high CBF in high-signal sagittal sinus regions (left) and the rest of the patient group (right). GM= gray matter, WM= white matter, 90th percentile SS = high-signal sagittal sinus regions.

	SCA (n=94)	Control (n=42)	*between-group differences*
Demographic Variables	Count (percentage) / Median (IQR)		
			Chi-Squared test / Mann-Whitney U test
Sex	46 Male (48.94%)	16 Male (38.10%)	X^2_1=0.97, p=0.32, φ=0.08
Age (yr)	16.67 (13.32 – 19.89)	17.33 (14.57 – 20.48)	U=2070.50, p=0.65, 95% CI=–1.45 – 2.32, r=0.04
Education Decile	5 (4 –7)	5 (4 – 6)	U=1899.50, p=0.72, 95% CI=-1.00 – 1.00, r=0.03
Hematological Variables			
Arterial oxygen content (CaO_2, mL/d)	11.79 (10.39 – 12.92)	17.69 (17.42 – 18.22)	**U=3934.00, p<0.0001***, 95% CI=5.76 – 6.98, r=0.79**
Radiological Variables			*Chi-Squared test / Mann-Whitney U test*
Silent Cerebral Infarction (SCI)	39 (41.49%)	4 (9.52%)	**X^2_1=12.28, p=0.0005***, φ=0.30**
SCI burden (1mm^3 voxels)	59 (33.5 – 149.5)	19 (15.0 – 35.5)	U=39.50, p=0.11, 95% CI= -401.00– 12.00, r= 0.14
Cognitive Variables	Mean (SD) / Median (IQR)		*Students t-test*
Intelligence quotient (IQ)	92.63 (13.44)	98.10 (12.00)	**$t_{88.7}$=2.35, p=0.02*, 95% CI=0.84 – 10.09, d=0.43**
Working memory index (WMI)	91.73 (13.69)	99.24 (13.48)	**$t_{81.0}$=2.97, p=0.004**, 95% CI=2.49 – 12.54, d=0.55**
Processing speed index (PSI)	89.49 (12.96)	97.55 (13.17)	**$t_{78.7}$= 3.29, p=0.001***, 95% CI=3.19 – 12.92, d=0.62**
Tower Completion Time	559.44 (147.44)	561.17 (151.88)	$t_{77.0}$= 0.06, p=0.95, 95% CI= -55.23 – 58.70, d=0.01
Tower Achievement	9 (8 – 10)	9 (8 – 11)	U=1941.0, p=0.56, 95% CI=-1.00 – 1.00, r=0.05
Hemodynamic Variables	Mean (SD) / Median (IQR)		*Students t-test / Mann-Whitney U test*
Gray Matter			
sTI ROI Size (1mm^3 voxels)	161274.83 (26963.09)	174110.29 (24302.00)	**$t_{88.5}$=2.72, p=0.008**, 95% CI=3466.40 – 22204.51, d=0.50**
mTI ROI Size (1mm^3 voxels)	153982.98 (24093.85)	170264.75 (23065.40)	**$t_{70.3}$=3.47, p=0.0009**, 95% CI=6920.40 – 25643.15, d=0.69**
sTI CBF (ml/100g/min)	54.06 (7.78)	42.52 (7.13)	**$t_{87.2}$=-8.39, p<0.0001***, 95% CI=-14.27 – -8.80, d=-1.55**
mTI CBF (ml/100g/min)	129.02 (22.26)	89.62 (11.83)	**$t_{110.8}$=-12.41, p<0.0001***, 95% CI=-45.69 – -33.11, d=-2.21**
mTI BAT (s)	0.72 (0.66 – 0.75)	0.82 (0.77 – 0.89)	**U=2473.00, p<0.0001***, 95% CI=0.08 – 0.14, r=0.53**
White Matter			
sTI ROI Size (1mm^3 voxels)	266598.2 (36692.39)	283498.50 (36483.00)	**$t_{80.9}$=2.47, p=0.015*, 95% CI=3285.87 – 30514.74, d=0.46**
mTI ROI Size (1mm^3 voxels)	263767 (237411.8 – 291997.5)	274221 (255279.0 – 304022.2)	**U= 1813.00, p=0.026*, 95% CI= 1886.00 – 28247.00, r=0.19**
sTI CBF (ml/100g/min)	31.25 (4.82)	22.91 (4.49)	**$t_{85.9}$=-9.68, p<0.0001***, 95% CI=-10.05 – -6.62, d=-1.79**
mTI CBF (ml/100g/min)	69.52 (11.99)	51.83 (7.46)	**$t_{102.4}$=-9.67, p<0.0001***, 95% CI=-21.32 – -14.06, d=-1.77**
mTI BAT (s)	0.96 (0.91 – 1.05)	1.15 (1.08 – 1.26)	**U=2515.00, p<0.0001***, 95% CI=0.14 – 0.23, r=0.55**
90th% percentile Sag. Sinus			
sTI ROI Size (1mm^3 voxels)	465.36 (118.22)	310.93 (69.86)	**$t_{122.5}$=-9.34, p<0.0001***, 95% CI= -187.15 – -121.71, d=-1.59**
mTI ROI Size (1mm^3 voxels)	455.77 (131.91)	311.06 (72.11)	**$t_{107.1}$=-7.46, p<0.0001***, 95% CI=-183.18 – -106.26, d=-1.36**
sTI CBF (ml/100g/min)	104.66 (80.06 – 145.60)	66.18 (57.03 – 78.93)	**U=636, p<0.0001***, 95% CI=-52.53 – -25.93, r=0.52**

mTI CBF (ml/100g/min)	253.77 (157.73 – 467.67)	145.83 (113.95 – 202.52)	**U=703, p< 0.0001***, 95% CI= -161.98 – -49.00, r=0.35**
mTI BAT (s)	1.17 (0.98 – 1.45)	1.01 (0.87 – 1.10)	**U=870, p=0.0025**, 95% CI= -0.32 – -0.065, r=0.26**

Table e1. Extended Sample demographics and cognitive performance. Values are summary and test statistics. Abbreviations: SCA = sickle cell anemia; sTI= single inflow time sequence, mTI = multi inflow time sequence, CBF = cerebral blood flow, BAT = bolus arrival time, *90th%ile Sag. Sinus* = high-signal regions of sagittal sinus, SD = standard deviation, IQR = interquartile range, 95% CI= 95% confidence interval
